# Catalytic Decarboxylation of Fatty Acids to Aviation Fuels over Nickel Supported on Activated Carbon

**DOI:** 10.1038/srep27820

**Published:** 2016-06-13

**Authors:** Jianghua Wu, Juanjuan Shi, Jie Fu, Jamie A. Leidl, Zhaoyin Hou, Xiuyang Lu

**Affiliations:** 1 Key Laboratory of Biomass Chemical Engineering of Ministry of Education, College of Chemical and Biological Engineering, Zhejiang University, Hangzhou 310027, China; 2 Key Laboratory of Applied Chemistry of Zhejiang Province, Department of Chemistry, Zhejiang University, Hangzhou 310028, China; 3 Department of Chemical and Biological Engineering, Western University, London N6A3K7, Canada

## Abstract

Decarboxylation of fatty acids over non-noble metal catalysts without added hydrogen was studied. Ni/C catalysts were prepared and exhibited excellent activity and maintenance for decarboxylation. Thereafter, the effects of nickel loading, catalyst loading, temperature, and carbon number on the decarboxylation of fatty acids were investigated. The results indicate that the products of cracking increased with high nickel loading or catalyst loading. Temperature significantly impacted the conversion of stearic acid but did not influence the selectivity. The fatty acids with large carbon numbers tend to be cracked in this reaction system. Stearic acid can be completely converted at 370 °C for 5 h, and the selectivity to heptadecane was around 80%.

Biofuel production research has been encouraged by the desire to displace fossil fuels and to mitigate CO_2_ emissions. Statistically, energy-related CO_2_ emissions from the transportation system has represented about 23% of the worldwide total^1^,^2^. The airline industry contributes about 2~3% of the global CO_2_ emissions and receives considerable attention^3^,^4^. Concern for the environment has promoted a strong incentive for the aviation industry to shift to the use of alternative fuel sources. The carbon of renewable feedstock comes from CO_2_ in the atmosphere by photosynthesis, which can equalize the CO_2_ emission from the use of renewable aviation fuels. Therefore, renewable aviation fuel was a long-term sustainable alternative for fossil fuels.

Aviation fuel is a special kind of liquid fuel that has a higher heating value and greater energy density than conventional fuels. It consists of C8–C16 alkanes, alkenes, and aromatic hydrocarbons^3^,^5^. Although bio-fuels such as bio-ethanol and bio-diesel have demonstrated their compatibility with vehicles, they cannot be added into aviation fuels since the components of aviation fuels have strict requirements.

Triglycerides, the main components of vegetable oils, animal fats, waste cooking oils and microalgae lipids, can produce straight chain alkanes, ranging from C8 to C18, that can be used for the production of aviation fuel^6^,^7^. At present, hydrodeoxygenation of triglycerides or free fatty acids is the main approach to producing aviation fuel from biomass^8^,^9^,^10^,^11^. The use of refined oil as the starting material and the high cost of hydrogen in the hydrodeoxygenation process, however, increase the cost of production^12^. Recently, decarboxylation of fatty acids and their derivatives without additional hydrogen was reported as a new approach for producing aviation fuel at a lower cost^13^,^14^,^15^. In our previous studies^15^,^16^,^17^, the decarboxylation behaviors, kinetics, and mechanisms of saturated and unsaturated fatty acids to alkanes over heterogeneous noble metal catalysts were determined. Noble metals such as Pd and Pt have been proved to be effective in catalyzing the decarboxylation of fatty acids and their derivatives^13^,^15^,^16^,^18^, but the high cost and low abundance of noble metal catalysts are serious constraints for large scale applications. Therefore, finding an inexpensive catalyst showing similar performance and durability is of great interest from an industrial standpoint.

Crocker and co-workers reported deoxygenation of triglycerides and fatty acids over supported nickel catalysts, triglycerides can be effectively into fuel-like hydrocarbon over Ni catalysts^19^,^20^,^21^,^22^, and the primary results are shown in Table 1 21,22. Ni/C afforded good yields of hydrocarbons in catalytic deoxygenation of stearic acid and triglycerides even in the absence of hydrogen. The selectivity to light hydrocarbons increases with the increase of unsaturation of the triglycerides, reflecting the greater reactivity of the unsaturated fatty acids towards cracking. Although Ni/C exhibited a promising activity for decarboxylation, the decarboxylation behaviors of saturated fatty acids over Ni/C catalysts, however, was not systematically studied.

In this article, several non-noble metal catalysts were prepared, and the catalytic activities for decarboxylation of stearic acid over non-noble catalysts were evaluated. The maintenance of 20% Ni/C and the products distribution were also studied. Thereafter, the effects of nickel loading, catalyst loading, temperature, and carbon number were investigated. The reaction regularity on the decarboxylation of stearic acid over Ni/C at different reaction conditions was discussed.

## Experimental Section

### Materials

Nickel nitrate hexahydrate (98%), aluminium nitrate hydrate(98%), cobalt nitrate hydrate(99%), cupric nitrate hydrate(99%) and zirconyl nitrate hydrate (99%) were purchased from Aladdin Industrial Corporation, Shanghai, China. 5% Pt/C and activated carbon were obtained from Sigma–Aldrich, USA, 40% Ni/SiO_2_ were purchased from Gansu Zhongkeyaoyuan Biological Engineering Co., Ltd, China. 20% Ni/Al_2_O_3_, 20% Cu/ZrO_2_, 20% Co/ZrO_2_ and 20% Ni/ZrO_2_ were synthesized in lab by the co-precipitation method. 10% Ni/C, 20% Ni/C and 30% Ni/C were synthesized in lab by the impregnation method. Stearic acid (>99%), lauric acid (>99%), myristic acid (>99%), palmitic acid (>99%), arachidic acid (>99%) and behenic acid (>99%) were all purchased from Sigma-Aldrich, USA. Acetone (analytic reagent grade) was obtained from Hangzhou Chemical Reagent Co., Ltd, China. The XRD results for all the catalysts were presented in Figure S1 or the section of “effects of nickel loadings on decarboxylation”.

### Catalyst characterization

#### N_2_ physisorption measurements

Surface areas and pore-size distribution were measured using nitrogen as a sorbate at 77 K in a static volumetric apparatus (Micromeritics ASAP2020). Samples were degassed prior to the measurements at 300 °C for 16 h. Specific surface areas were calculated using the Brunauer-Emmett-Teller (BET) equation and calculation of pore sizes and pore volumes followed the method of Barret-Joyner-Halenda (BJH). According to implemented software routines, the Halsey thickness equation was used for relating the thickness of the adsorbed layer to the relative pressure. All calculations were based on the adsorption model.

#### X-Ray diffraction (XRD)

X-Ray diffraction technique was used for identifying the phases present in the synthesized samples and calculating the crystallite size of Ni by the Scherrer equation. XRD patterns were recorded on a PANalytical Empyrean 200895, using Ni filtered Cu Kα radiation (λ = 0.154 nm) as a source (current intensity, 30 mA; voltage, 40 kV) and X-celerator detector. The samples were scanned in the 2θ range of 10–80°.

The elemental composition of the reduced nickel supported catalysts was determined by inductively coupled plasma atomic emission spectroscopy (ICP-AES) using Leeman Labs Inc, USA (Model: PS 3000 UV) ICP-AES analyzer.

### Experimental procedure

**Figure 1:**
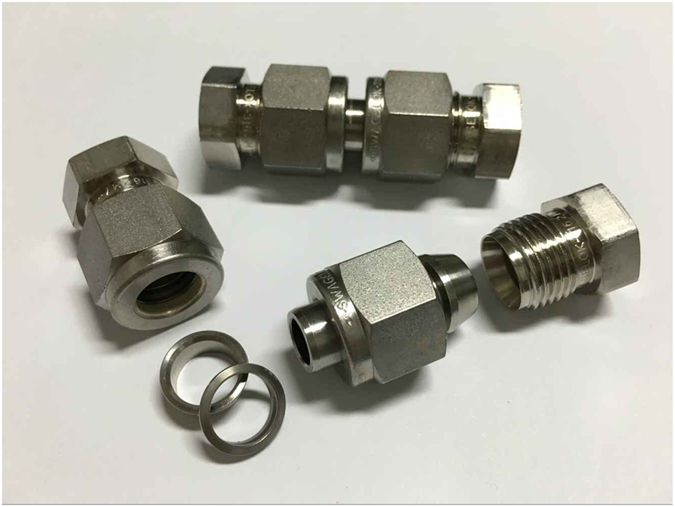
Photo of micro batch reactor and its parts.

The decarboxylation of fatty acids was carried out in a micro batch reactor (1.67 mL volume), which was assembled from one 3/8-inch tube and two 3/8-inch caps purchased from Swagelok, USA. The photo of the micro batch reactor and its parts is shown in Fig. 1. A certain amount of reactant and catalyst were added to the reactor. Thereafter, the sealed reactor was placed in a fluidized sand bath (Techne SBL-2) which was heated to the desired reaction temperature. After the desired reaction time had elapsed, the reactor was put into water to quench the reaction. The reaction mixture in the reactor was rinsed and diluted in a 10 mL volumetric flask with acetone. Then the diluted solution was filtered for analysis.

### Product analysis

The samples were analyzed with a gas chromatograph (GC, Agilent 7890A) equipped with a 30 m × 0.25 mm × 0.33 mm HP-5 capillary column and a flameionization detector. 1 μL sample was injected into the GC with a split ratio of 10:1, and the carrier gas (nitrogen) flow rate was 11 mL/min. The temperature of the injector and detector were 280 and 300 °C, respectively. The oven temperature program consisted of a 4 min soak at 40 °C followed by a 10 °C/min ramp up to 280 °C, which was held for 5 min. The reaction products were identified by fragmentation patterns from an Agilent 5970 Mass Spectrometric(MS) detector and by matching gas chromatograph retention times with known standards. Quantitative analysis was performed using calibration curves for each compound of interest.

Reactant molar conversions were calculated as the number of moles of reactant consumed divided by the initial number of moles of reactant loaded into the reactor. Selectivity was calculated as the number of moles of product recovered divided by the number of moles of reactant that had reacted (i.e., yield/conversion). Uncertainties reported are standard deviations, which were determined by replicating experiments. Each data point represents the mean result from at least three independent experiments.

## Results and Discussion

### Catalytic activity and maintenance

**Figure 2:**
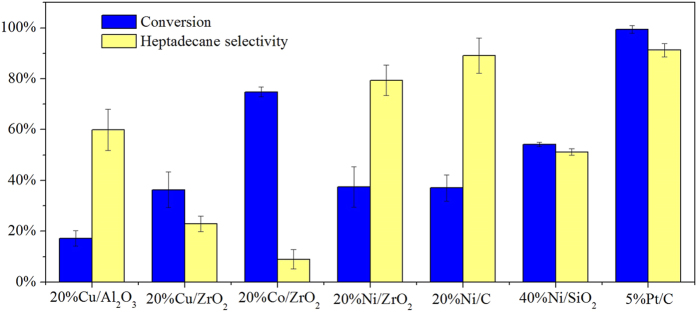
Evaluation of non-noble metal catalysts for catalytic activity of decarboxylation. Reaction conditions: T = 330 °C, time = 5 h, catalyst loading = 30 mg, stearic acid loading = 0.176 mmol.

The catalytic activities for decarboxylation of stearic acid over different non-noble catalysts were evaluated, shown in Fig. 2. The reaction was carried out at 330 °C for 5 h. The loading of stearic acid was 0.176 mmol, and the loading of catalyst was 30 mg. The error bars represent standard deviations of three replicate experiments. The conversions of stearic acid over Cu/ZrO_2_, Co/ZrO_2_ and Ni/ZrO_2_ were 36.3%, 74.8% and 37.4% respectively, and the selectivities of heptadecane were 22.9%, 8.9% and 79.4% respectively. Although the conversion of stearic acid over Co/ZrO_2_ was the highest, the selectivity of heptadecane over Co/ZrO_2_ was the lowest, leading a very low yield of heptadecane (6.7%), which was lower than those over Cu/ZrO_2_ (8.3%) and Ni/ZrO_2_ (29.7%). Synthetically, the catalytic performance of nickel was superior to copper and cobalt for decarboxylation. The conversions of stearic acid over Ni/C, Ni/SiO_2_ and Ni/ZrO_2_ were 37.1%, 54.1% and 37.4% respectively, and the selectivities of heptadecane were 89.1%, 51.2% and 79.4% respectively. Although the conversion of stearic acid over Ni/SiO_2_ was higher than those over Ni/C and Ni/ZrO_2_, the selectivity of heptadecane over Ni/SO_2_ was much lower than those over Ni/C and Ni/ZrO_2_, leading to lower yield. The conversions of stearic acid over Ni/C and Ni/ZrO_2_ were very close, but the selectivity of heptadecane over Ni/C was higher than that over Ni/ZrO_2_. Synthetically, activated carbon was better as a support than ZrO_2_ and SiO_2_. Stearic acid could be completely converted over 5% Pt/C within 2 h at 330 °C, and the selectivity of heptadecane was 91%. The conversion efficiency of stearic acid over Ni/C was 7.4% per hour, while that of stearic acid over Pt/C was 49.7% per hour. Although the conversion efficiency of Ni/C for decarboxylation was not as good as Pt/C, the selectivities to heptadecane over Ni/C and Pt/C were quite close, meaning that heptadecane can be selectively obtained over Ni/C by longer time or higher temperature. The critical point for the potential use of nickel as a catalyst is that the price of nickel is much cheaper than platinum, which would significantly lower the cost of production although higher temperature or longer reaction time are required.

### Effects of nickel loadings on decarboxylation

**Figure 3:**
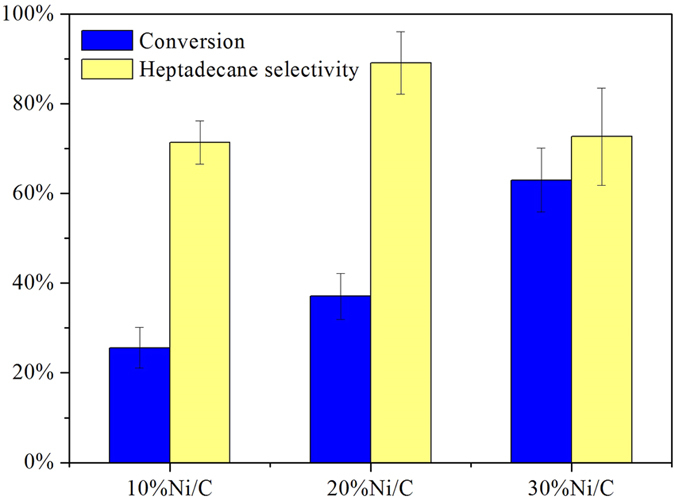
Conversion of stearic acid and selectivity to heptadecane over catalysts with different loadings. Reaction Conditions: T = 330 °C, time = 5 h, catalyst loading = 30 mg, stearic acid loading = 0.176 mmol.

The decarboxylation of stearic acid was examined over three Ni/C catalysts with different nickel loadings (10%, 20% and 30%) under the same experimental conditions (Temperature = 330 °C, time = 5 h, catalyst loading = 30 mg, stearic acid loading = 0.176 mmol). The effect of nickel loading on the conversion of stearic acid and the selectivity to heptadecane was shown in Fig. 3. As observed from the figure, the conversion of stearic acid increased with increasing nickel loading on activated carbon. The selectivity to heptadecane over 20% Ni/C was much higher than 10% Ni/C and 30% Ni/C.

The products from the decarboxylation of stearic acid over 20% Ni/C and 30% Ni/C were identified by GC/MS. Except heptadecane, other products such as decane, undecane, dodecane, tridecane, tetradecane, pentadecane, hexadecane, heptadecene, octadecane, and octadecanol were also detected. We defined the total areas of the detected peaks as 100%, and obtained the relative percentage (area of peak/total area of peaks) of each product, and the product distribution is shown in Table 2. The product distribution indicates that cracking and hydrodeoxygenation occurred during the decarboxylation. Compared to the sample catalyzed by 20% Ni/C, more alkanes with small carbon number were detected in the sample catalyzed by 30% Ni/C (decane: 0.8%; undecane: 1.2%; dodecane: 1.6%). Moreover, the overall yield of cracking products (alkanes with smaller carbon numbers than heptadecane) over 30% Ni/C and 20% Ni/C was 21.3% and 10.4% respectively, indicating that 30% Ni/C tends to catalyze cracking compared to 20% Ni/C.

**Figure 4:**
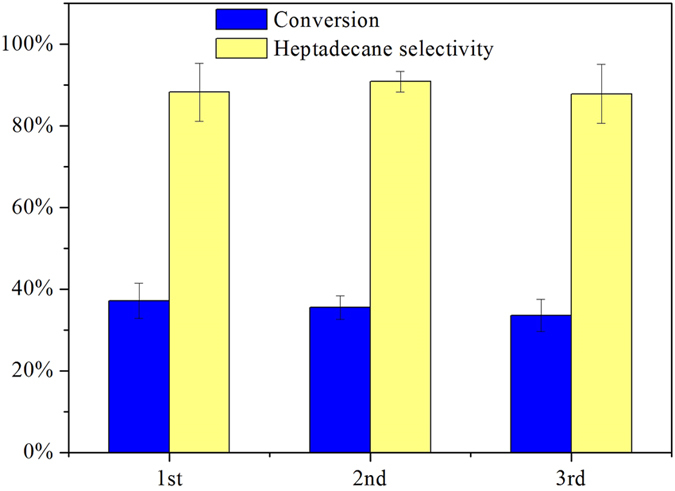
Conversion of stearic acid and selectivity to heptadecane over recycled 20% Ni/C. Reaction conditions: T = 330 °C, time = 5 h, catalyst loading = 30 mg, stearic acid loading = 0.176 mmol.

To study the reusability of Ni/C catalyst, spent catalysts of previous experiments were first separated by filtration under vacuum and then washed thoroughly with acetone followed by water multiple times. The filtered catalyst was then dried in a forced air oven at 110 °C overnight and then calcined at 500 °C for 4 h under the flow of nitrogen in a tubular furnace. The activity maintenance of 20% Ni/C was evaluated at 330 °C for 5 h with a catalyst loading of 30 mg and 0.176 mmol of reactant, shown in Fig. 4. The conversions of stearic acid, yields, and selectivities to heptadecane over fresh Ni/C (1^st^ use), catalyst used once previously (2^nd^ use), and catalyst used twice previously (3^rd^ use) were almost the same. The selectivity remained around 90%, keeping stable in a solvent free system.

**Figure 5:**
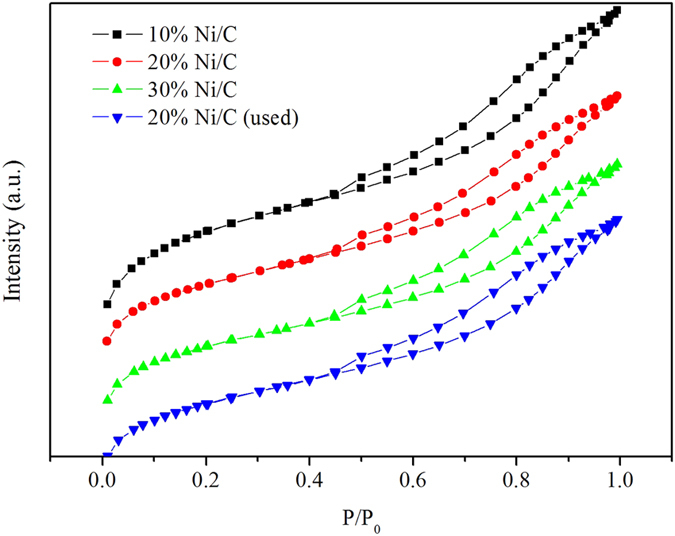
N2 adsorption-desorption plot for Ni/C catalysts.

**Figure 6:**
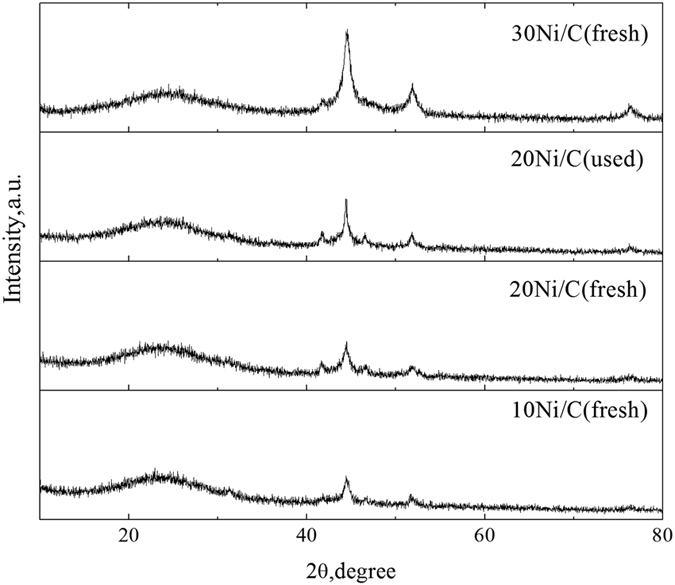
XRD results for the Ni/C catalysts with different nickel loadings.

The N_2_ adsorption-desorption isotherms of 10% Ni/C, 20% Ni/C, 30% Ni/C and 20% Ni/C (used) were shown in Fig. 5. The isotherms of these catalysts exhibited similar sharps, indicating that the structure of these catalysts were not different, even after used. The surface area and the porosity structure of Ni/C samples were summarized in Table 3. The TEM results of Ni/C at low magnification, high magnification, and the distribution of Ni particle size are shown in Figure S2. The average pore sizes of these catalysts were around 3.5 nm, and the mean Ni particle size was 11.7 nm from the TEM results (Figure S2c), while the mean Ni particle size was 13.7 nm from the XRD results. The surface areas and pore volumes decreased as the nickel loadings increased, indicating excessive Ni particles might block a certain amount of pores of activated carbon and decrease the pore volume. The average pore sizes for the catalysts were almost the same. X-ray diffraction (XRD) further investigated the crystalline Ni particles supported on activated carbon (Fig. 6). The prominent peak at 26° was recorded and ascribed to the reflection of amorphous carbon support. Apart from the characteristic peaks of carbon support, the peaks at 44.5°, 51.8° and 76.4° can be assigned to Ni (JCPDS 04–0850). According to the XRD data, the peak signals of Ni-based particles gradually enhanced with increased Ni loadings, indicating the fluctuation of Ni particles.

### Effects of catalyst loading

**Figure 7:**
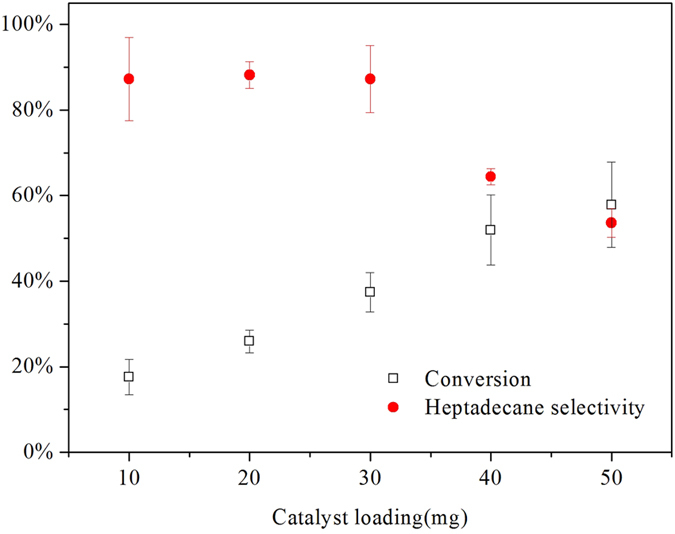
Conversion of stearic acid and selectivity to heptadecane for decarboxylation of stearic acid over 20% Ni/C at different catalyst loadings. Reaction conditions: T = 330 °C, time = 5 h, stearic acid loading = 0.176 mmol.

The effects of catalyst loading on conversion of stearic acid and the selectivity for heptadecane were studied for five different 20% Ni/C catalyst loadings at 330 °C for 5 h, as shown in Fig. 7. The error bars represent standard deviations of three replicate experiments. The decarboxylation reaction of stearic acid was carried out without any catalyst, and no conversion of stearic acid was observed after 5 h. In Fig. 5, the conversion of stearic acid increased continuously from 17.6% to 57.8% as the catalyst loading increased from 10 to 50 mg. The selectivity of heptadecane remained around 90% as the catalyst loading increased from 10 to 30 mg. When the loading amount of 20% Ni/C was higher than 30 mg, the selectivity of heptadecane decreased, suggesting excess amount of catalysts was not favorable for the decarboxylation.

### Effects of temperature

**Figure 8:**
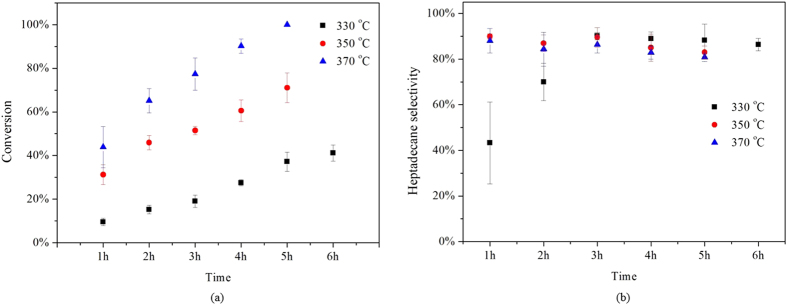
Conversion of stearic acid and selectivity to heptadecane for decarboxylation of stearic acid over 20% Ni/C at different temperatures. Reaction conditions: catalyst loading = 30 mg, stearic acid loading = 0.176 mmol.

Figure 8 shows the time course of the decarboxylation of stearic acid over 20% Ni/C at three different temperatures, 330, 350, 370 °C. It was found that the conversion of stearic acid increased with prolonged reaction time, and continuously increased with increasing reaction temperature. At 370 °C, complete conversion of stearic acid was achieved in 5 h. The selectivity for heptadecane at these temperatures and reaction times ranged around 90%. These results indicate that the 20% Ni/C in the decarboxylation of stearic acid has a high selectivity for heptadecane, even at high temperatures.

### Decarboxylation of fatty acids with varying carbon numbers

**Figure 9:**
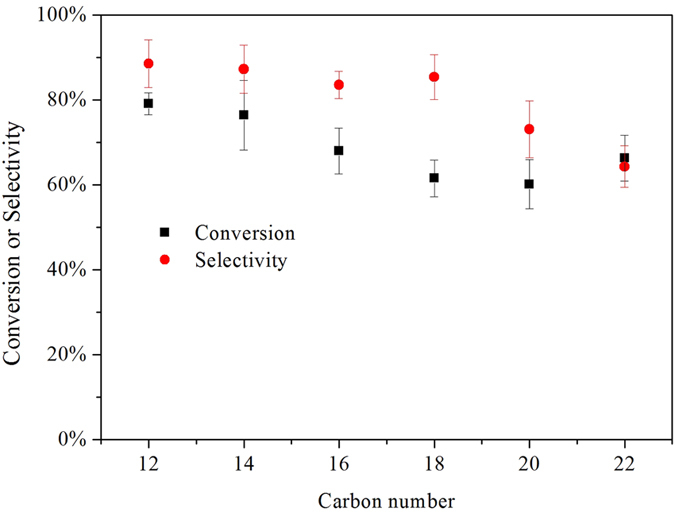
Conversion of fatty acids and selectivity to alkanes for decarboxylation over 20% Ni/C. Reaction conditions: T = 350 °C, time = 4 h, catalyst loading = 30 mg, fatty acid loading = 0.20 mmol.

The decarboxylation of different fatty acids (lauric acid, myristic acid, palmitic acid, stearic acid, arachidic acid and behenic acid) with the same molar ratio of reactant loading to catalyst loading were studied at 350 °C for 4 h as shown in Fig. 9. These results suggested that different fatty acids with varying carbon numbers also underwent decarboxylation over Ni/C. The conversions of lauric acid, myristic acid, palmitic acid, stearic acid, arachidic acid and behenic acid were 79.1%, 76.4%, 67.9%, 61.5%, 60.2% and 66.3% respectively, and the selectivities to the corresponding alkanes were 88.5%, 87.2%, 83.5%, 85.4%, 73.1% and 64.3% respectively. The conversions of the fatty acids with smaller carbon numbers were higher than those with larger carbon numbers at the same reaction condition. The acidity decreased in the sequence of lauric acid, myristic acid, palmitic acid and stearic acid^23^, consistent with the decrease sequence of fatty acid conversion. It indicates that the acidity of fatty acids might facilitate the decarboxylation. Moreover, the fatty acids with large carbon numbers had lower selectivity to the corresponding alkane than those with small carbon numbers, indicating the fatty acids with large carbon numbers tend to be cracked in this reaction system.

## Conclusion

Ni/C catalysts were prepared and exhibited excellent activity and maintenance for decarboxylation. Stearic acid can be completely converted at 370 °C for 5 h, and the selectivity to heptadecane was around 80%. Excessive Ni particles might block a certain amount of pores of activated carbon and decrease the pore volume. The products of cracking increased with high nickel loading or catalyst loading. Temperature significantly impacted the conversion of stearic acid but did not influence the selectivity. The fatty acids with large carbon numbers tend to be cracked in this reaction system.

## Additional Information

**How to cite this article**: Wu, J. *et al*. Catalytic Decarboxylation of Fatty Acids to Aviation Fuels over Nickel Supported on Activated Carbon. *Sci. Rep.* **6**, 27820; doi: 10.1038/srep27820 (2016).

## Figures and Tables

**Table 1:** Reported results of catalytic decarboxylation of fatty acids over Ni-based catalysts^21^,^22^.

Catalyst	Feed	Gas	Temperature(°C)	Time (h)	Conversion (%)	Selectivity to C_10-17_ (%)	Selectivity to C_17_ (%)
20% Ni/C	Stearic acid	N_2_	300	1.5	19	50	26
20% Ni/C	Stearic acid	10% H_2_/N_2_	300	1.5	64	77	51
20% Ni/C	Stearic acid	H_2_	300	1.5	80	88	81
20% Ni/Al_2_O_3_	Stearic acid	N_2_	300	1.5	9	48	38
20% Ni/Al_2_O_3_	Stearic acid	10% H_2_/N_2_	300	1.5	80	85	67
20% Ni/Al_2_O_3_	Stearic acid	H_2_	300	1.5	81	84	57
Ni-Al LDH	Stearic acid	N_2_	300	1.5	10	53	13
Ni-Al LDH	Stearic acid	10% H_2_/N_2_	300	1.5	42	43	30
Ni-Al LDH	Stearic acid	H_2_	300	1.5	73	61	52
20% Ni/C	Tristearin	N_2_	360	6	81	75	30
20% Ni/C	Tristearin	10% H_2_/N_2_	360	6	88	75	53
20% Ni/C	Tristearin	H_2_	360	6	>99	77	55
20% Ni/Al_2_O_3_	Tristearin	N_2_	355	6	86	58	31
20% Ni/Al_2_O_3_	Tristearin	10% H_2_/N_2_	355	6	96	71	36
20% Ni/Al_2_O_3_	Tristearin	H_2_	355	6	>99	70	7
Ni-Al LDH	Tristearin	N_2_	355	6	88	70	40
Ni-Al LDH	Tristearin	10% H_2_/N_2_	355	6	90	72	40
Ni-Al LDH	Tristearin	H_2_	355	6	81	86	69

**Table 2:** Product distribution for stearic acid decarboxylation over 20% and 30% Ni/C.

Retention time	Compound	Area % (20%Ni/C)	Area % (30%Ni/C)
10.040	decane	/	0.8
12.045	undecane	/	1.2
13.835	dodecane	/	1.6
15.464	tridecane	1.0	2.2
16.978	tetradecane	1.7	3.2
18.393	pentadecane	2.9	5.0
19.727	hexadecane	4.8	7.2
20.813	heptadecene	2.1	2.3
21.056	heptadecane	73.1	72.2
22.911	octadecane	4.0	4.2
25.596	octadecanol	5.0	/
26.151	stearic acid	5.5	/

**Table 3:** Nickel loading, surface area and pore size for fresh 10%, 20%, 30% Ni/C catalysts and used 20% Ni/C catalysts.

Catalyst	Nickel (wt.%)	BET surface area (m^2^/g)	Pore volume (cm^3^/g)	Average pore size (nm)
10% Ni/C(fresh)	9.2	722	0.642	3.6
20% Ni/C(fresh)	18.9	651	0.566	3.5
30% Ni/C(fresh)	27.4	624	0.550	3.4
20% Ni/C(used)	17.8	565	0.519	3.7
